# Meta-analysis of the efficacy and safety of Finerenone in diabetic kidney disease

**DOI:** 10.1097/MD.0000000000047098

**Published:** 2026-01-23

**Authors:** Kai Chen, Wei Shao, Huaxiong Zhang

**Affiliations:** a Department of Endocrinology, The First People’s Hospital of Jiande, Hangzhou, Zhejiang Province, China.

**Keywords:** diabetic nephropathies, Finerenone, meta-analysis, renal function

## Abstract

**Objective:**

This study is aimed to systematically evaluate the efficacy and safety of Finerenone (BAY 94-8862) in the treatment of diabetic kidney disease.

**Method:**

Databases including PubMed, Embase, and Web of science were searched for randomized controlled trials that assessed Finerenone in patients with diabetic kidney disease and were published up to January 7, 2025. The quality evaluation of literatures and meta-analysis were performed using RevMan 5.4 software and independently operated by 2 experimenters.

**Results:**

Seven randomized controlled trials involving 33,455 patients were included. The efficacy outcomes were the ratio of urine albumin creatine ratio at posttreatment versus at baseline (mean difference: −0.30; 95% confidence interval [CI; −0.32, −0.27]; *P* < .00001; *I*^2^ = 0%), the primary composite outcome (hazard ratio [HR]: 0.81; 95% CI [0.76, 0.87]; *P* < .00001; *I*^2^ = 13%), the secondary composite outcome (HR: 0.82; 95% CI [0.74, 0.91]; *P* = .0001; *I*^2^ = 0%), kidney failure (HR: 0.76; 95% CI [0.70, 0.83]; *P* < .00001; *I*^2^ = 0%), and the risk of end stage kidney disease (HR: 0.87; 95% CI [0.77, 0.99]; *P* = .03; *I*^2^ = 46%). The safety outcome was the incidence of adverse events (risk ratio: 1.00; 95% CI [0.99, 1.00]; *P* = .38; *I*^2^ = 0%) and the number of patients suffering from hyperkalemia (risk ratio: 2.19; 95% CI [2.04, 2.34]; *P* < .00001; *I*^2^ = 0%).

**Conclusion:**

Results of meta-analysis showed that Finerenone could significantly reduce urine albumin creatine ratio levels. It has a protective effect on kidney, delaying the progression of chronic kidney disease and reducing the risk of end stage kidney disease. Furthermore, there was no difference in the risk of overall adverse events.

## 1. Introduction

Diabetic kidney disease (DKD) is an important microvascular complication of diabetes, which is considered one of the main causes of end stage kidney disease (ESKD).^[1]^ During the development of DKD, the filtration function of the glomerulus is continuously impaired, gradually leading to renal failure and ultimately developing into ESKD. Causing huge health and economic burdens for both patients and society, DKD may lead to renal failure and need dialysis or kidney transplantation. In recent decades, the prevalence of type 2 diabetes has been rising.^[2]^ About 40% of type 2 diabetes mellitus patients will develop into DKD. Although the mechanism of DKD is still unclear, researchers have revealed that DKD may be related to renal fibrosis and proteinuria generation, etc.^[3]^

Various of treatments have been developed in DKD, including inhibitors of the nuclear factor kappa-B pathway,^[4]^ inhibitors of the transforming growth factor-β pathway, and anti-inflammatory cytokines.^[5]^ Finerenone (BAY-94-8862) is a new nonsteroidal selective mineralocorticoid receptor antagonist, which has strong anti-inflammatory and anti-fibrosis effects.^[6]^ While ensuring high selectivity of receptors, it also greatly avoids the risk of steroid induced adverse events (such as hyperkalemia and gonadal suppression).^[7]^ In clinical trials, it has been proved that it can reduce the ratio of urinary albumin to creatinine in chronic kidney disease (CKD) patients,^[8,9]^ and it has been proved in FIGARO-DKD and FIDELIO-DKD studies that it can reduce the heart rate in patients with advanced CKD and type 2 diabetes mellitus vascular incidence rate and mortality.^[3,10]^

The existing evidence for the treatment of mineralocorticoid receptor antagonists in DKD remains unclear. Previous meta-analyses related to Finerenone were mostly about cardiovascular diseases. Currently, a few meta-analysis studies on Finerenone for the treatment of DKD. However, due to their early publication, the number of literatures included was limited. In this meta-analysis of randomized, placebo-controlled trials, the efficacy and safety of Finerenone in the treatment of DKD was systematically evaluated. The efficacy outcomes included urine albumin creatinine ratio (UACR) at posttreatment versus at baseline, the primary composite outcome, the secondary composite outcome, kidney failure, and the incidence of ESKD. The safety outcome was the incidence of adverse events and the number of patients suffering from hyperkalemia.

## 2. Method

This study was approved by the Ethics Committee of The First People’s Hospital of Jiande. This study was conducted according to the Preferred Reporting Items for Systematic Reviews and Meta-Analyses Extension Statement for Reporting of Systematic Reviews Incorporating Network Meta-analyses of Health Care Interventions: Checklist and Explanations. The protocol of this study was registered with International Prospective Register of Systematic Reviews (CRD420250651179).

### 2.1. Search strategy

This meta-analysis was conducted to evaluate the efficacy and safety of Finerenone in the treatment of DKD. Databases including PubMed, Embase, and Web of science were searched for randomized controlled trials (RCT) that assessed Finerenone in the treatment of DKD and were published up to January 7, 2025. The keywords used are as follows: “Diabetic Nephropathies,” “Nephropathies, Diabetic,” “Nephropathy, Diabetic,” “Diabetic Kidney Disease,” “Diabetic Kidney Diseases,” “Kidney Disease, Diabetic,” “Kidney Diseases, Diabetic,” “Diabetic Nephropathy,” “Diabetic Glomerulosclerosis,” “Glomerulosclerosis, Diabetic,” “Intracapillary Glomerulosclerosis,” “Kimmelstiel-Wilson Disease,” “Kimmelstiel Wilson Disease,” “Nodular Glomerulosclerosis,” “Glomerulosclerosis, Nodular,” “Kimmelstiel-Wilson Syndrome,” “Kimmelstiel Wilson Syndrome,” “Syndrome, Kimmelstiel-Wilson,” “finerenone,” “BAY 94-8862,” “kerendia.”

### 2.2. Inclusion and exclusion criteria

Eligible studies were meeting criteria as follow: patients (≥18 years old) with type 2 diabetes and CKD treated with a reninangiotensin system (RAS) inhibitor (angiotensinconverting–enzyme inhibitor or angiotensinreceptor blocker) at the maximum dose on the manufacturer’s label that did not cause unacceptable side effects; the intervention measure is oral administration of any dose of finerenone; at least one interested outcome was reported; RCTs published in English. The exclusion criteria were as follows: patients with a serum potassium concentration ≥ 4.8 mmol/L; patients receiving renal replacement therapy; animal experiments; meta-analysis, review, case report, conference, and letter. Two reviewers independently screened the studies, and discrepancies were resolved through discussion or consultation with a third reviewer. Inter-reviewer agreement was assessed using Cohen’s κ coefficient, which demonstrated substantial consistency.

### 2.3. Efficacy and safety outcomes

Efficacy outcomes: the ratio of UACR at posttreatment versus at baseline; hazard ratio (HR) for the primary composite outcome (a sustained decrease of at least 40% in the estimated glomerular filtration rate (eGFR) from baseline); HR for the secondary composite outcome (a sustained decrease of at least 57% in the eGFR from baseline; HR for kidney failure (defined as a sustained eGFR of <15 mL/min per 1.73 m^2^ of body-surface area); HR for ESKD (defined as the initiation of long-term dialysis or kidney transplantation). Safety outcomes: the number of adverse events; the number of patients suffering from hyperkalemia.

### 2.4. Data extraction

A data extraction sheet (based on the Cochrane Consumers and Communication Review Group’s data extraction template) was developed. Two evaluators (Kai, Wei) extracted data independently. Information was extracted from each included trial on: characteristics of trial participants (including age, gender, and serum potassium levels) and the trials inclusion and exclusion criteria; types of intervention (including doses, duration and frequency of Finerenone); outcomes, length of follow up and adverse events reported.

### 2.5. Risk of bias in included RCTs

Two reviewers (Kai, Wei) assessed and validated the quality of included trials independently according to Cochrane Handbook for Systematic Reviews of Interventions. Disagreements were resolved by consulting a third reviewer Huaxiong. Potential selection bias (random sequence generation and allocation concealment), performance bias (blinding of participants and personnel), attrition bias (incomplete outcome data), reporting bias (selective reporting) and other sources of bias were assessed in this part.

### 2.6. Statistical analysis

Meta-analyses and assessment of risk of bias in included RCTs were performed by using the Cochrane Collaboration software RevMan5.4. Binary data was presented using risk ratio (RR) with 95% confidence interval (CI). Continuous data was presented as mean difference (MD) with 95% CI. HR was presented as HR with 95% CI. Statistical heterogeneity among trials was assessed by Cochrane chi-square (*χ*^2^) test with an α of 0.05 used for statistical significance and quantified by Cochrane-*I*² test with 95% CI. The random effect model was used if statistical heterogeneity existed (*I*^2^ > 50%); the fixed effect model was used if the trials were basically homogeneous (*I*^2^ ≤ 50%).

## 3. Results

### 3.1. Baseline characteristics of the studies

**Figure 1.**
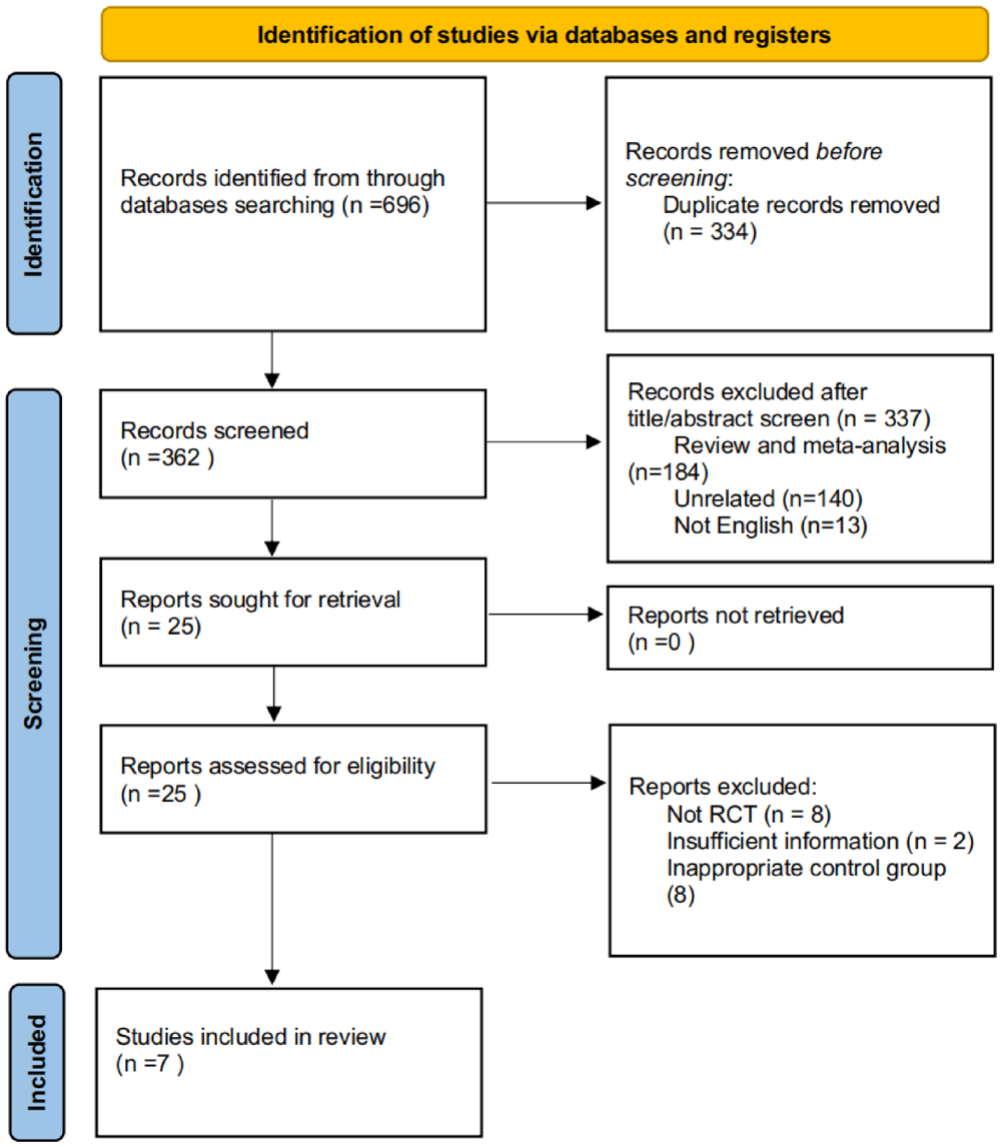
Process flowchart of literature screening.

A total of 696 studies were retrieved from the databases mentioned above, and 7 RCT involving 33,455 patients were included^[11-17]^ (Fig. 1). The sample size of each study ranged from 96 to 13,026, while the average ages of patients varied from 62.4 to 67. Of all studies, 4 provided data on the ratio of UACR at posttreatment versus at baseline, 4 reported HR for the primary composite outcome, 5 submitted data on HR for the second composite outcome, 4 reported HR for kidney failure, 4 reported HR for kidney failure, 4 reported HR for ESKD, 7 reported the number of adverse events and 6 reported the number of patients suffering from hyperkalemia (Table 1). Although 7 RCTs were included in the meta-analysis, not all trials reported every outcome indicator; therefore, the number of studies included in each pooled analysis varied.

**Table 1:** Characteristics of studies included.

Study	Country	Age		Sample		Intervention		Outcomes	Conflict of interest
		T	C	T	C	T	C		
Katayama^[11]^	Japan	62.4 (9.80)	66.7 (9.02)	84	12	Finerenone (1.25–10 mg)	Placebo	(1)(6)(7)	–
Bakris^[12]^	United States	–	–	6519	6507	Finerenone (10 or 20 mg)	Placebo	(3)(5)(6)(7)	Yes
Sarafidis^[13]^	United States	67 (9)	67 (9)	440	450	Finerenone (10 or 20 mg)	Placebo	(2)(3)(5)(6)(7)	Yes
Barkris^[14]^	United States	65.7 (9.2)	65.4 (8.9)	2840	2833	Finerenone (10 or 20 mg)	Placebo	(1)(2)(3) (5)(6)(7)	Yes
Barkris^[15]^	United States	64.3 (25.08)	63.26 (8.68)	727	94	Finerenone (1.25–20 mg)	Placebo	(1)(6)(7)	Yes
Pitt^[16]^	United States	64.1 (9.7)	64.1 (10.0)	3686	3666	Finerenone (10 or 20 mg)	Placebo	(1)(2)(3) (4) (5)(6)(7)	Yes
Ruilope ^[17]^	United States	65.43 (8.96)	65.68 (9.18)	2830	2839	Finerenone (10 or 20 mg)	Placebo	(2)(3)(6)(7)	Yes

C = control group, eGFR = estimated glomerular filtration rate, ESKD = end stage kidney disease, HR = hazard ratio, T = treatment group, UACR = urine albumin creatinine ratio, –  = not mentioned.

(1) The ratio of UACR at posttreatment versus at baseline.

(2) Hazard ratio (HR) for the primary composite outcome (a sustained decrease of at least 40% in the estimated glomerular filtration rate (eGFR) from baseline).

(3) HR for the secondary composite outcome (a sustained decrease of at least 57% in the eGFR from baseline.

(4) HR for kidney failure (defined as a sustained eGFR of <15 mL/min per 1.73 m^2^ of body-surface area).

(5) HR for ESKD (defined as the initiation of long-term dialysis or kidney transplantation).

(6) The number of adverse events.

(7) The number of patients suffering from hyperkalemia.

### 3.2. Risk of bias in included RCTs

**Figure 2.**
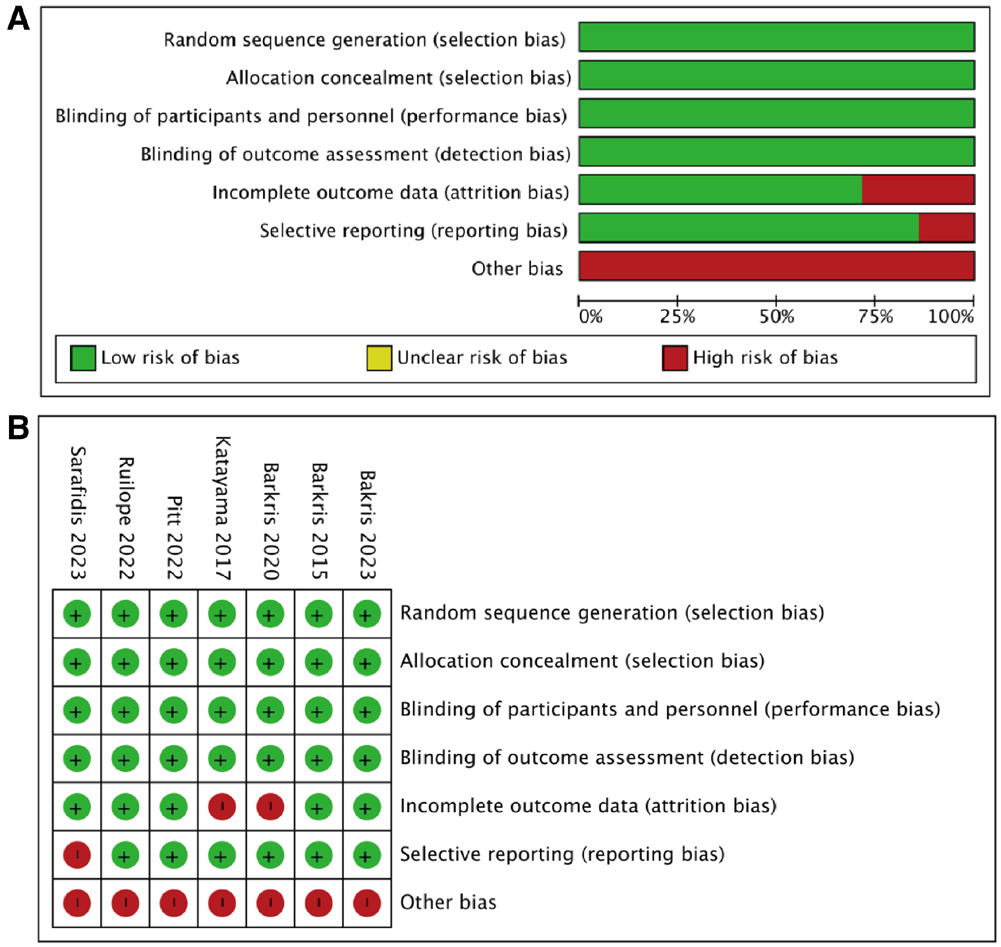
Risk of bias. (A) Review authors’ judgments about each risk of bias item presented as percentages across all included studies. (B) Review authors’ judgments about each risk of bias item for each included study.

The risk of bias was assessed with the Cochrane Handbook for Systematic reviews of interventions, and the trials showed a low to moderate risk of bias (Fig. 2). All experiments showed “random” and “double-blind.” All RCTS were considered low-risk in terms of random sequence generation, allocation concealment, selective reporting, blinding of participants and personnel, and bias due to blinding in outcome assessment. Two studies were considered unclear risk of bias of incomplete outcome data, which were missing data on UACR at posttreatment versus at baseline. All 7 RCT were considered potentially risky as they have received support from Bayer, the development company of Finerenone.

### 3.3. The ratio of UACR

**Figure 3.**
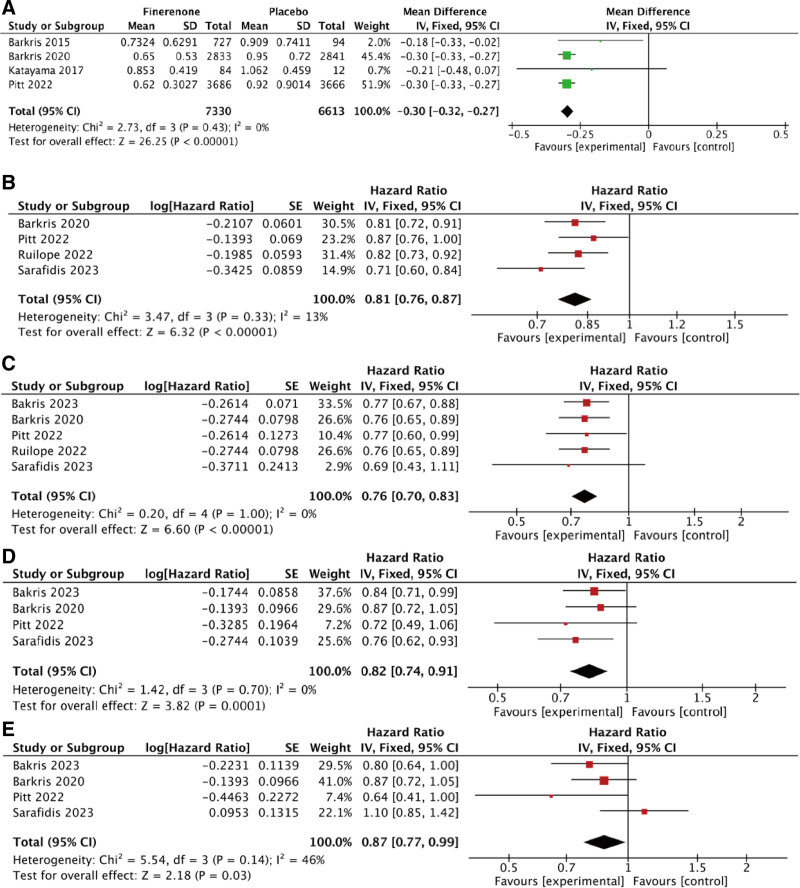
The meta analysis of efficacy outcomes. Forest plot of comparison: (A) Finerenone vs Placebo, outcome: LS mean ratio of UACR; (B) Finerenone vs Placebo, outcome: HR for primary composite outcome; (C) Finerenone vs Placebo, outcome: HR for secondary composite kidney outcome; (D) Finerenone vs Placebo, outcome: HR for kidney failure; (E) Finerenone vs Placebo, outcome: HR for ESKD. ESKD = end stage kidney disease, HR = hazard ratio, LS = least-squares, UACR = urine albumin creatinine ratio.

**Figure 4.**
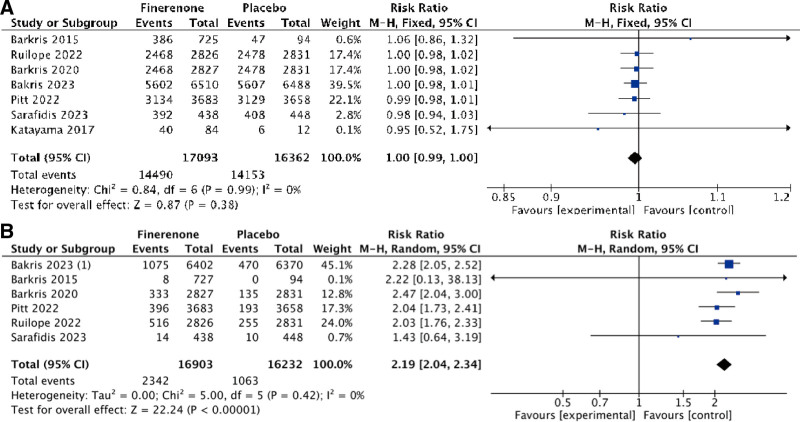
The meta analysis of safety outcomes. Forest plot of comparison: (A) Finerenone vs Placebo, outcome: number of AE; (B) 2 Finerenone vs Placebo, outcome: Hyperkalemia. AE = adverse event.

Six studies reported UACR. Four of them presented it in terms of least-squares (LS) mean ratio, while 2 of them presented in terms of HR. A fixed-effect model was utilized for analyzing the LS mean ratio of UACR from baseline to follow-up time. Results were summarized in Figure 4. It was indicated that compared to the placebo group, the LS mean ratio of UACR in the finerenone group was significantly reduced, with a MD of (MD: −0.30; 95% CI [−0.32, −0.27]; *P* < .00001; *I*^2^ = 0%), indicating a statistically significant difference (Fig. 3A).

### 3.4. HR for the composite outcome

The primary composite outcome was defined as a sustained decrease of at least 40% in eGFR from baseline or death from renal causes. Four RCTS reported HR for the primary composite outcome. A fixed-effect model was utilized for analyzing. Finerenone could significantly decrease the HR of the primary composite outcome of kidney (HR: 0.81; 95% CI [0.76, 0.87]; *P* < .00001; *I*^2^ = 13%; Fig. 3B). The secondary composite outcome was defined as a sustained decrease of at least 57% in the eGFR from baseline. Five RCTS reported HR for the secondary composite outcome of kidney. A fixed-effect model was applied, finerenone could significantly decrease the HR of the secondary composite outcome of kidney (HR: 0.76; 95% CI [0.70, 0.83]; *P* < .00001; *I*^2^ = 0%; Fig. 3C).

### 3.5. HR for kidney failure

Kidney failure was defined as a sustained eGFR of <15 mL/min per 1.73 m^2^ of body-surface area. For the 4 distinct studies reported kidney failure, a forest plot for generic inverse variance was created. To evaluate HR for kidney failure, a fixed effects model was utilized as a result of low heterogeneity (*χ*^2^ = 1.42, df = 3, *I*^2^ = 0%), indicating that the individual findings of each study demonstrated a significant influence on HR for kidney failure (HR: 0.82; 95% CI [0.74, 0.91]; *P* = .0001; *I*^2^ = 0%; Fig. 3D).

### 3.6. HR for ESKD

ESKD was defined as the initiation of long-term dialysis or kidney transplantation. For the 4 studies reported data on ESKD, a forest plot for generic inverse variance was created. To evaluate HR for ESKD, a fixed effects model was utilized. The heterogeneity study is relatively high (*I*^2^ = 46%, *P* = .14) but within an acceptable range (*I*^2^ < 50%, *P* > .10). Finerenone demonstrated a significant influence on HR for ESKD (HR: 0.87; 95% CI [0.77, 0.99]; *P* = .03; *I*^2^ = 46%; Fig. 3E).

### 3.7. Adverse events

All of the 7 studies reported data on adverse events that occurred during the treatment period such as serious adverse events, acute kidney injury and hyperkalemia, etc. The incidence of adverse events that occurred during the treatment period was similar in the finerenone and placebo groups. Finerenone did not demonstrate a significant influence on adverse events (RR: 1.00; 95% CI [0.99, 1.00]; *P* = .38; *I*^2^ = 0%; Fig. 4A).

### 3.8. Hyperkalemia

Hyperkalemia was a pathological condition characterized by a serum potassium concentration exceeding 5.5 mmol/L, primarily due to impaired kidney function, excessive potassium intake, or the use of certain medications. Six studies reported data on number of patients suffering from hyperkalemia, showing minimal variation among the studies (*I*^2^ = 0%, *P* = .42; Fig. 4B). A fixed effect model was employed, and Finerenone and placebo differed statistically significantly in their risk of hyperkalemia (RR: 2.19; 95% CI [2.04, 2.34]; *P* < .00001; *I*^2^ = 0%).

## 4. Discussion

In this study, we conducted a meta-analysis to compare the efficacy and safety of Finerenone in the treatment of DKD. The study included 7 RCT involving 33,455 patients. For patients, most of indexes were consistent such as age, sex and intervention, etc. Therefore, meta-analysis was derived based on fixed effects model. Meta-analysis of efficacy outcomes indicated that Finerenone has significant efficacy in reducing UACR, reducing the risk of significant eGFR decline, and reducing the risk of kidney failure.

UACR is a sensitive index to evaluate renal filtration function, which is often elevated in patients with diabetes nephropathy.^[18]^ The increase of UACR in patients with DKD is a multifactorial process. Long term hyperglycemia is the core driving force of this pathological change. By promoting the formation of glycation end products, exacerbating oxidative stress reactions, and activating inflammatory signaling pathways, it directly damages the structure and function of glomerular endothelial cells, basement membrane, and podocytes, leading to impaired glomerular filtration barrier and increased albumin filtration.^[19,20]^ Compared with Placebo, Finerenone has a significant effect in reducing UACR levels (MD: −0.30; 95% CI [−0.32, −0.27]; *P* < .00001; *I*^2^ = 0%), which has similar to previous meta-analyses.^[21]^

EGFR is an important indicator used to evaluate renal filtration function.^[22]^ Meta-analysis of HR for the primary and secondary composite outcomes indicated that patients received finerenone had a lower risk of a primary (HR: 0.81; 95% CI [0.76, 0.87]; *P* < .00001; *I*^2^ = 13%)and secondary (HR: 0.76; 95% CI [0.70, 0.83]; *P* < .00001; *I*^2^ = 0%) outcome event than those who received placebo. Finerenone is a nonsteroidal selective mineralocorticoid receptor antagonist that exerts strong anti-inflammatory and anti-fibrotic effects by directly blocking the excessive activation of mineralocorticoid receptors. By slowing down the inflammation and fibrosis process of the kidneys, Finerenone protects the structure and function of the glomeruli, reducing damage and sclerosis of the glomeruli, maintaining the effective filtration area and filtration function of the glomeruli, and therefore reducing the risk of a significant decrease in eGFR.^[18]^

Similarly, Finerenone reduces the activation of pro-inflammatory and pro-fibrotic signaling pathways in the kidneys, which helps alleviate the inflammatory response of the kidneys, reducing fibrosis and sclerosis of kidney tissue. During the development of DKD, persistent inflammation and fibrosis are the key factors that lead to the gradual loss of renal function and ultimately to kidney failure.^[23]^ Meta-analysis of HR for kidney failure indicated that patients received finerenone had a lower risk of kidney failure (defined as a sustained eGFR of <15 mL/min per 1.73 m^2^ of body-surface area; HR: 0.76; 95% CI [0.70, 0.83]; *P* = .0001; *I*^2^ = 0%). Although finerenone was associated with a higher risk of hyperkalemia (RR 2.19), the clinical severity was generally low, with few cases leading to treatment discontinuation. This finding is consistent with previous studies on combination therapy with SGLT2 inhibitors or statins, where the incidence of severe hyperkalemia remained low and manageable.

ESKD is 5-stage of CKD, indicating severe impairment of kidney function and almost complete loss of its normal function.^[24]^ In 7 studies included, 4 studies reported hazard risk of ESKD. Meta-analysis results of ESKD demonstrated that the impact of Finerenone on the HR of ESKD is significant. As reported in Bakris, 2020, the early reduction in albuminuria, early separation of the Kaplan–Meier curves for the key secondary outcome, and modest blood-pressure reduction that the protective effect of finerenone on the kidneys may be partially mediated by the natriuretic mechanism.^[14]^ However, hemodynamic effects were also seen in trials of dual RAS blockade that did not show efficacy. Preclinical data showed that the kidney benefits of finerenone were associated with potent antiinflammatory and antifibrotic effects through inhibition of overactivation of the mineralocorticoid receptor.^[25]^

There was no significant difference in the overall incidence of adverse events between the Finerenone groups and the placebo group, and there was no relevant increase in adverse events across finerenone dosages.^[11]^ Hyperkalemia, which refers to a high concentration of potassium ions in the blood, usually above 5.5 mmol/L, is a common side effect of Finerenone.^[26]^ Finerenone treatment is expected to increase serum potassium concentration as it inhibits the potassium excretion function of the kidney, leading to the retention of potassium ions in the body. As shown in results of meta-analysis, hyperkalemia was more frequent with finerenone than with placebo. However, the clinical impact of hyperkalemia was minimal. There were no deaths due to hyperkalemia, and the incidence of hyperkalemia leading to permanent treatment discontinuation was low in all patients, markedly lower than in trials of dual RAS blockade (4.8% with combination therapy with a direct renin inhibitor and an angiotensin-converting enzyme inhibitor or angiotensin receptor blocker and 9.2% with dual angiotensin-converting enzyme inhibitor and angiotensin receptor blocker therapy^[25]^).

## 5. Conclusion

Overall, results of meta-analysis showed that the hazard risk of the kidney composite outcome, the secondary composite outcome, kidney failure and ESKD was reduced with Finerenone compared with placebo. Finerenone could significantly reduce UACR levels, has a protective effect on kidney, delay the progression of CKD, and reduce the risk of ESKD. Although finerenone was associated with a higher overall risk of hyperkalemia than placebo, discontinuation of the trial regimen due to hyperkalemia was infrequent in patients who received finerenone. Furthermore, there was no difference in the risk of overall adverse events.

## Author contributions

**Conceptualization:** Kai Chen, Wei Shao, Huaxiong Zhang.

**Data curation:** Kai Chen, Wei Shao, Huaxiong Zhang.

**Formal analysis:** Kai Chen, Wei Shao, Huaxiong Zhang.

**Funding acquisition:** Kai Chen, Wei Shao, Huaxiong Zhang.

**Investigation:** Kai Chen, Wei Shao, Huaxiong Zhang.

**Writing – original draft:** Kai Chen, Huaxiong Zhang.

**Writing – review & editing:** Kai Chen, Huaxiong Zhang.
